# Supplementary material for: Vitamin D Modified DSS-Induced Colitis in Mice via STING Signaling Pathway
Source: Biology (Basel). 2025 Jun 18;14(6):715. doi: 10.3390/biology14060715 (PMC12190092; doi:10.3390/biology14060715)
Supplement: Supplementary file 1 [file biology-14-00715-s001.zip › Supplementary Materials S1: 16s-report of gut microbiota/Groups/diff_analysis/metagenomeROrder/figure/boxplot/top10.pdf]

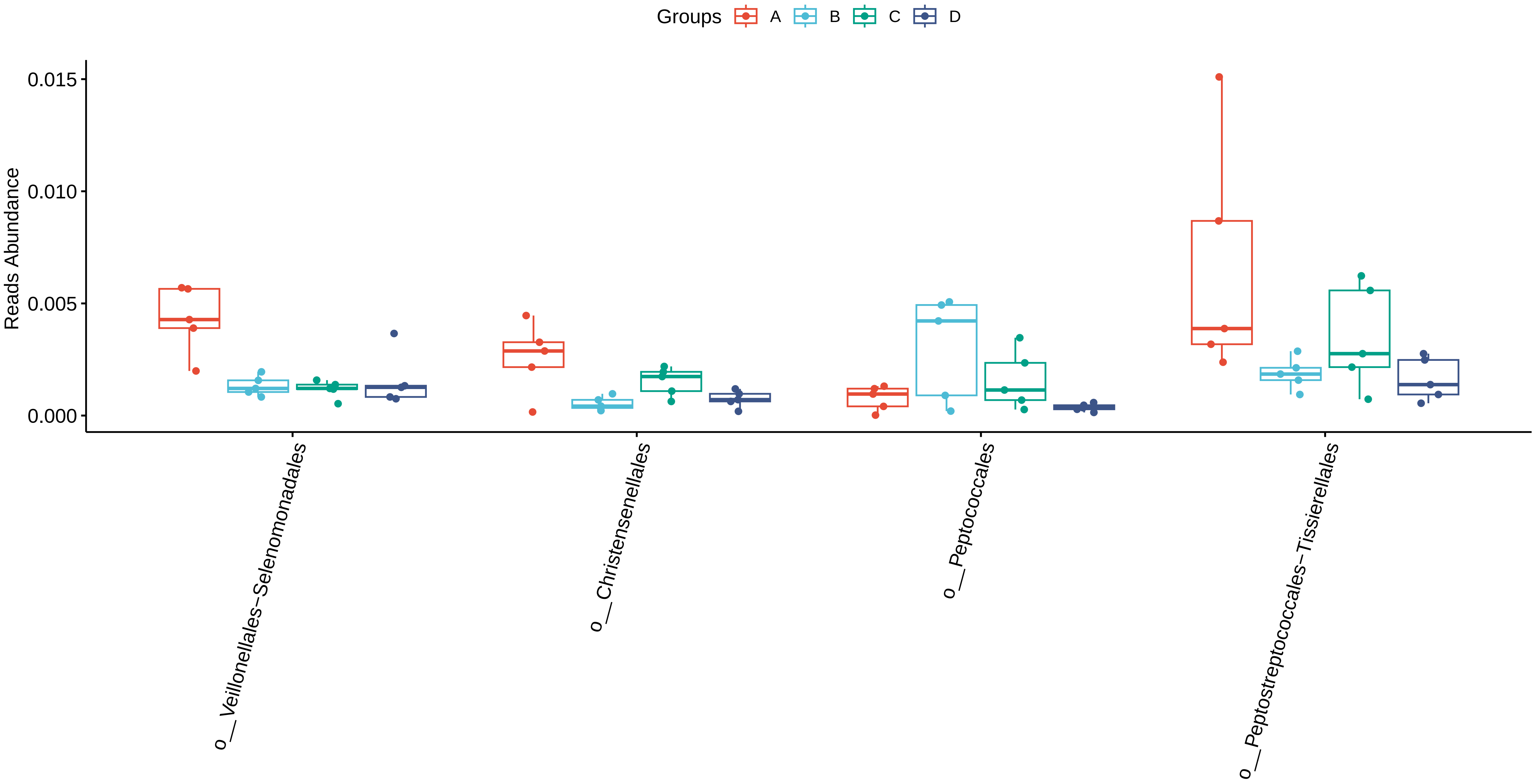
Groups
A
B
C
D
Reads Abundance
0.015
0.010
0.005
0.000
o__Veillonellales–Selenomonadales
o__Christensenellales
o__Peptococcales
o__Peptostreptococcales–Tissierellales
